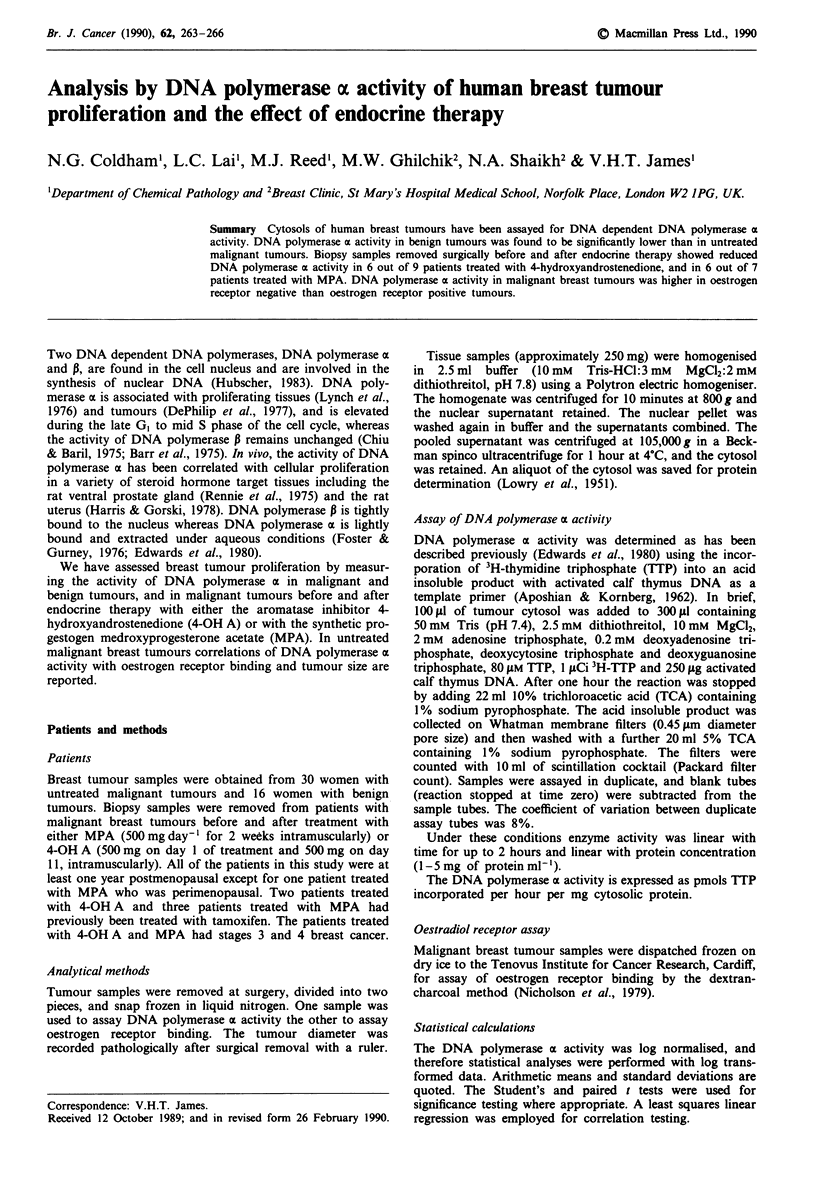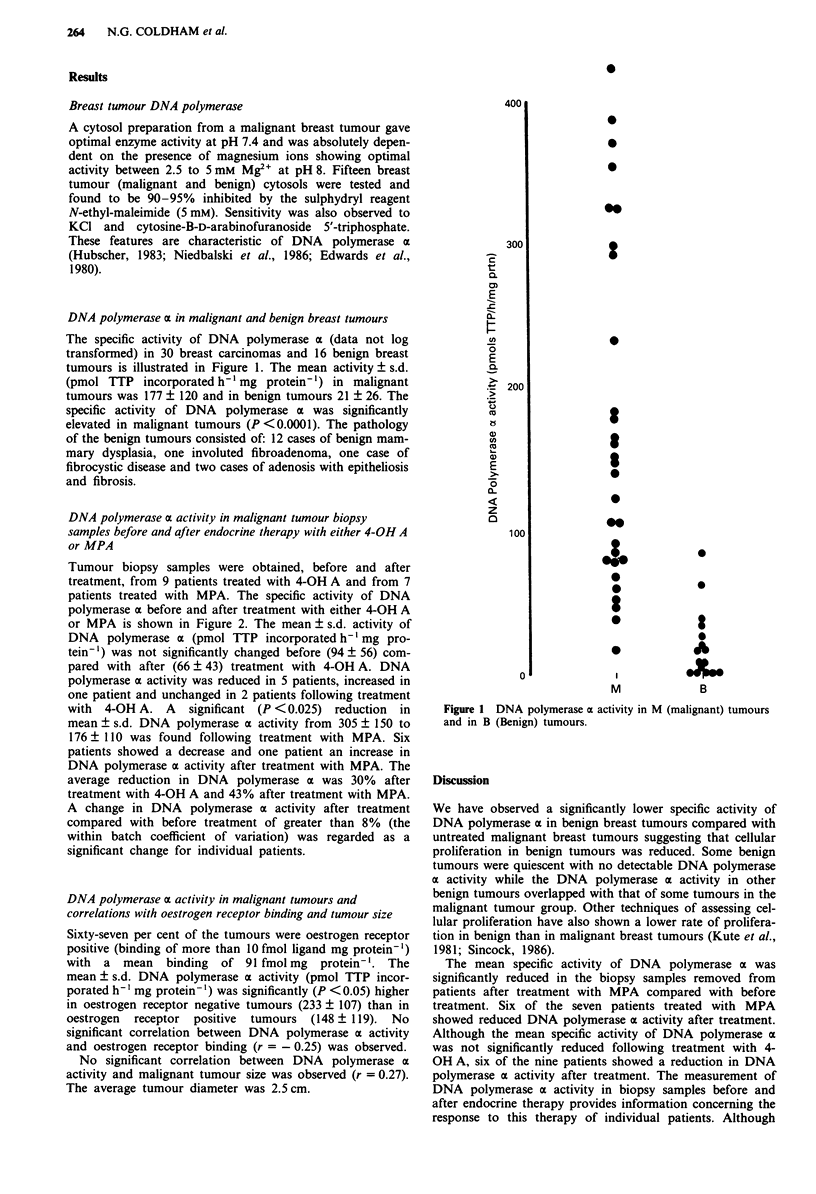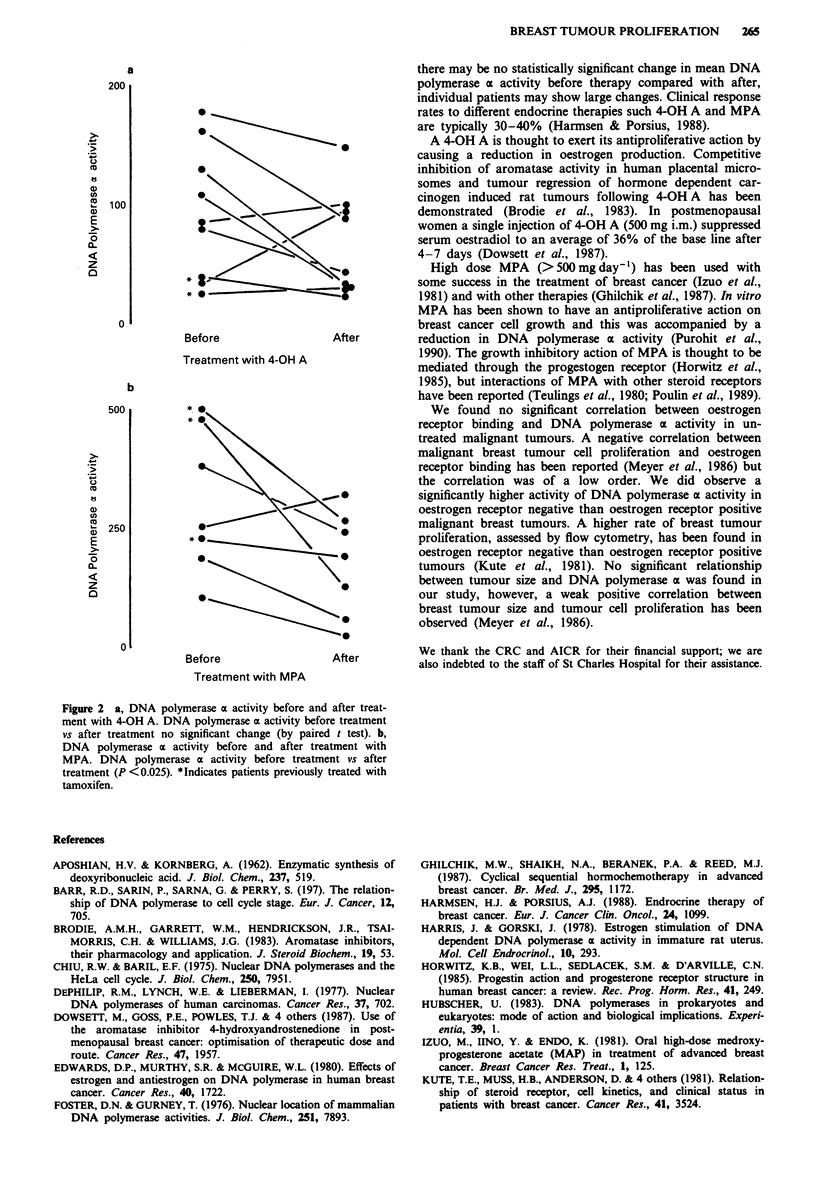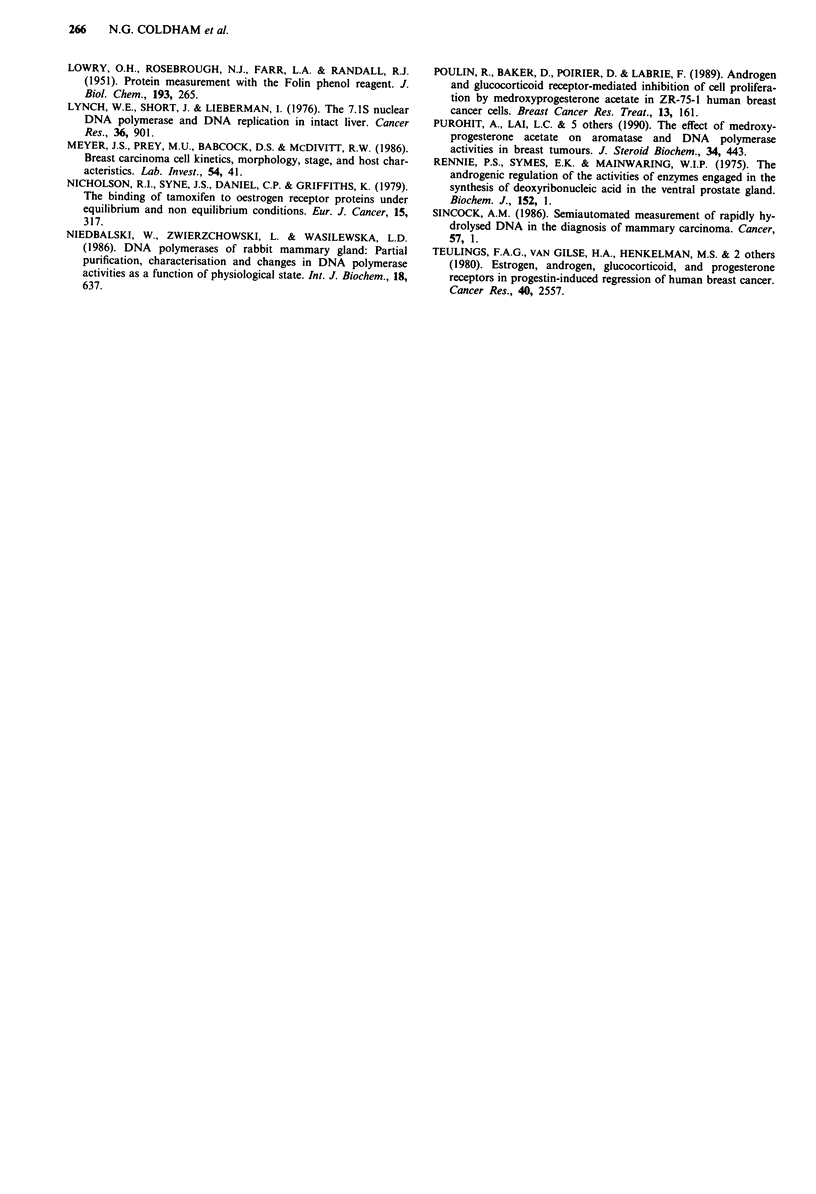# Analysis by DNA polymerase alpha activity of human breast tumour proliferation and the effect of endocrine therapy.

**DOI:** 10.1038/bjc.1990.273

**Published:** 1990-08

**Authors:** N. G. Coldham, L. C. Lai, M. J. Reed, M. W. Ghilchik, N. A. Shaikh, V. H. James

**Affiliations:** Department of Chemical Pathology, St Mary's Hospital Medical School, London, UK.

## Abstract

Cytosols of human breast tumours have been assayed for DNA dependent DNA polymerase alpha activity. DNA polymerase alpha activity in benign tumours was found to be significantly lower than in untreated malignant tumours. Biopsy samples removed surgically before and after endocrine therapy showed reduced DNA polymerase alpha activity in 6 out of 9 patients treated with 4-hydroxyandrostenedione, and in 6 out of 7 patients treated with MPA. DNA polymerase alpha activity in malignant breast tumours was higher in oestrogen receptor negative than oestrogen receptor positive tumours.


Br. J. Cancer (1990), 62, 263-266 © Macmillan Press Ltd., 1990

### Analysis by DNA polymerase α activity of human breast tumour proliferation and the effect of endocrine therapy

N.G. Coldham[1], L.C. Lai[1], M.J. Reed[1], M.W. Ghilchik[2], N.A. Shaikh[2] & V.H.T. James[1]

[1]*Department of Chemical Pathology and* [2]*Breast Clinic, St Mary's Hospital Medical School, Norfolk Place, London W2 1PG, UK.*

**Summary** Cytosols of human breast tumours have been assayed for DNA dependent DNA polymerase α activity. DNA polymerase α activity in benign tumours was found to be significantly lower than in untreated malignant tumours. Biopsy samples removed surgically before and after endocrine therapy showed reduced DNA polymerase α activity in 6 out of 9 patients treated with 4-hydroxyandrostenedione, and in 6 out of 7 patients treated with MPA. DNA polymerase α activity in malignant breast tumours was higher in oestrogen receptor negative than oestrogen receptor positive tumours.

Two DNA dependent DNA polymerases, DNA polymerase α and β, are found in the cell nucleus and are involved in the synthesis of nuclear DNA (Hubscher, 1983). DNA polymerase α is associated with proliferating tissues (Lynch *et al.*, 1976) and tumours (DePhilip *et al.*, 1977), and is elevated during the late $G_1$ to mid S phase of the cell cycle, whereas the activity of DNA polymerase β remains unchanged (Chiu & Baril, 1975; Barr *et al.*, 1975). *In vivo*, the activity of DNA polymerase α has been correlated with cellular proliferation in a variety of steroid hormone target tissues including the rat ventral prostate gland (Rennie *et al.*, 1975) and the rat uterus (Harris & Gorski, 1978). DNA polymerase β is tightly bound to the nucleus whereas DNA polymerase α is lightly bound and extracted under aqueous conditions (Foster & Gurney, 1976; Edwards *et al.*, 1980).

We have assessed breast tumour proliferation by measuring the activity of DNA polymerase α in malignant and benign tumours, and in malignant tumours before and after endocrine therapy with either the aromatase inhibitor 4-hydroxyandrostenedione (4-OH A) or with the synthetic progestogen medroxyprogesterone acetate (MPA). In untreated malignant breast tumours correlations of DNA polymerase α activity with oestrogen receptor binding and tumour size are reported.

### Patients and methods

### *Patients*

Breast tumour samples were obtained from 30 women with untreated malignant tumours and 16 women with benign tumours. Biopsy samples were removed from patients with malignant breast tumours before and after treatment with either MPA (500 mg $day^{-1}$ for 2 weeks intramuscularly) or 4-OH A (500 mg on day 1 of treatment and 500 mg on day 11, intramuscularly). All of the patients in this study were at least one year postmenopausal except for one patient treated with MPA who was perimenopausal. Two patients treated with 4-OH A and three patients treated with MPA had previously been treated with tamoxifen. The patients treated with 4-OH A and MPA had stages 3 and 4 breast cancer.

### *Analytical methods*

Tumour samples were removed at surgery, divided into two pieces, and snap frozen in liquid nitrogen. One sample was used to assay DNA polymerase α activity the other to assay oestrogen receptor binding. The tumour diameter was recorded pathologically after surgical removal with a ruler.

Tissue samples (approximately 250 mg) were homogenised in 2.5 ml buffer (10 mM Tris-HCl:3 mM $MgCl_2$:2 mM dithiothreitol, pH 7.8) using a Polytron electric homogeniser. The homogenate was centrifuged for 10 minutes at 800 *g* and the nuclear supernatant retained. The nuclear pellet was washed again in buffer and the supernatants combined. The pooled supernatant was centrifuged at 105,000 *g* in a Beckman spinco ultracentrifuge for 1 hour at 4°C, and the cytosol was retained. An aliquot of the cytosol was saved for protein determination (Lowry *et al.*, 1951).

### *Assay of DNA polymerase α activity*

DNA polymerase α activity was determined as has been described previously (Edwards *et al.*, 1980) using the incorporation of $^3$H-thymidine triphosphate (TTP) into an acid insoluble product with activated calf thymus DNA as a template primer (Aposhian & Kornberg, 1962). In brief, 100 μl of tumour cytosol was added to 300 μl containing 50 mM Tris (pH 7.4), 2.5 mM dithiothreitol, 10 mM $MgCl_2$, 2 mM adenosine triphosphate, 0.2 mM deoxyadenosine triphosphate, deoxycytosine triphosphate and deoxyguanosine triphosphate, 80 μM TTP, 1 μCi $^3$H-TTP and 250 μg activated calf thymus DNA. After one hour the reaction was stopped by adding 22 ml 10% trichloroacetic acid (TCA) containing 1% sodium pyrophosphate. The acid insoluble product was collected on Whatman membrane filters (0.45 μm diameter pore size) and then washed with a further 20 ml 5% TCA containing 1% sodium pyrophosphate. The filters were counted with 10 ml of scintillation cocktail (Packard filter count). Samples were assayed in duplicate, and blank tubes (reaction stopped at time zero) were subtracted from the sample tubes. The coefficient of variation between duplicate assay tubes was 8%.

Under these conditions enzyme activity was linear with time for up to 2 hours and linear with protein concentration (1-5 mg of protein $ml^{-1}$).

The DNA polymerase α activity is expressed as pmols TTP incorporated per hour per mg cytosolic protein.

### *Oestradiol receptor assay*

Malignant breast tumour samples were dispatched frozen on dry ice to the Tenovus Institute for Cancer Research, Cardiff, for assay of oestrogen receptor binding by the dextran-charcoal method (Nicholson *et al.*, 1979).

### *Statistical calculations*

The DNA polymerase α activity was log normalised, and therefore statistical analyses were performed with log transformed data. Arithmetic means and standard deviations are quoted. The Student's and paired *t* tests were used for significance testing where appropriate. A least squares linear regression was employed for correlation testing.

Correspondence: V.H.T. James.
Received 12 October 1989; and in revised form 26 February 1990.

### Results

*Breast tumour DNA polymerase*

A cytosol preparation from a malignant breast tumour gave optimal enzyme activity at pH 7.4 and was absolutely dependent on the presence of magnesium ions showing optimal activity between 2.5 to 5 mM $Mg^{2+}$ at pH 8. Fifteen breast tumour (malignant and benign) cytosols were tested and found to be 90–95% inhibited by the sulphydryl reagent *N*-ethyl-maleimide (5 mM). Sensitivity was also observed to KCl and cytosine-B-D-arabinofuranoside 5′-triphosphate. These features are characteristic of DNA polymerase α (Hubscher, 1983; Niedbalski *et al.*, 1986; Edwards *et al.*, 1980).

*DNA polymerase α in malignant and benign breast tumours*

The specific activity of DNA polymerase α (data not log transformed) in 30 breast carcinomas and 16 benign breast tumours is illustrated in Figure 1. The mean activity ± s.d. (pmol TTP incorporated $h^{-1}$ mg protein$^{-1}$) in malignant tumours was 177 ± 120 and in benign tumours 21 ± 26. The specific activity of DNA polymerase α was significantly elevated in malignant tumours ($P < 0.0001$). The pathology of the benign tumours consisted of: 12 cases of benign mammary dysplasia, one involuted fibroadenoma, one case of fibrocystic disease and two cases of adenosis with epitheliosis and fibrosis.

*DNA polymerase α activity in malignant tumour biopsy samples before and after endocrine therapy with either 4-OH A or MPA*

Tumour biopsy samples were obtained, before and after treatment, from 9 patients treated with 4-OH A and from 7 patients treated with MPA. The specific activity of DNA polymerase α before and after treatment with either 4-OH A or MPA is shown in Figure 2. The mean ± s.d. activity of DNA polymerase α (pmol TTP incorporated $h^{-1}$ mg protein$^{-1}$) was not significantly changed before (94 ± 56) compared with after (66 ± 43) treatment with 4-OH A. DNA polymerase α activity was reduced in 5 patients, increased in one patient and unchanged in 2 patients following treatment with 4-OH A. A significant ($P < 0.025$) reduction in mean ± s.d. DNA polymerase α activity from 305 ± 150 to 176 ± 110 was found following treatment with MPA. Six patients showed a decrease and one patient an increase in DNA polymerase α activity after treatment with MPA. The average reduction in DNA polymerase α was 30% after treatment with 4-OH A and 43% after treatment with MPA. A change in DNA polymerase α activity after treatment compared with before treatment of greater than 8% (the within batch coefficient of variation) was regarded as a significant change for individual patients.

*DNA polymerase α activity in malignant tumours and correlations with oestrogen receptor binding and tumour size*

Sixty-seven per cent of the tumours were oestrogen receptor positive (binding of more than 10 fmol ligand mg protein$^{-1}$) with a mean binding of 91 fmol mg protein$^{-1}$. The mean ± s.d. DNA polymerase α activity (pmol TTP incorporated $h^{-1}$ mg protein$^{-1}$) was significantly ($P < 0.05$) higher in oestrogen receptor negative tumours (233 ± 107) than in oestrogen receptor positive tumours (148 ± 119). No significant correlation between DNA polymerase α activity and oestrogen receptor binding ($r = -0.25$) was observed.

No significant correlation between DNA polymerase α activity and malignant tumour size was observed ($r = 0.27$). The average tumour diameter was 2.5 cm.

**Figure 1** DNA polymerase α activity in M (malignant) tumours and in B (Benign) tumours.

### Discussion

We have observed a significantly lower specific activity of DNA polymerase α in benign breast tumours compared with untreated malignant breast tumours suggesting that cellular proliferation in benign tumours was reduced. Some benign tumours were quiescent with no detectable DNA polymerase α activity while the DNA polymerase α activity in other benign tumours overlapped with that of some tumours in the malignant tumour group. Other techniques of assessing cellular proliferation have also shown a lower rate of proliferation in benign than in malignant breast tumours (Kute *et al.*, 1981; Sincock, 1986).

The mean specific activity of DNA polymerase α was significantly reduced in the biopsy samples removed from patients after treatment with MPA compared with before treatment. Six of the seven patients treated with MPA showed reduced DNA polymerase α activity after treatment. Although the mean specific activity of DNA polymerase α was not significantly reduced following treatment with 4-OH A, six of the nine patients showed a reduction in DNA polymerase α activity after treatment. The measurement of DNA polymerase α activity in biopsy samples before and after endocrine therapy provides information concerning the response to this therapy of individual patients. Although

**Figure 2** **a**, DNA polymerase α activity before and after treatment with 4-OH A. DNA polymerase α activity before treatment *vs* after treatment no significant change (by paired *t* test). **b**, DNA polymerase α activity before and after treatment with MPA. DNA polymerase α activity before treatment *vs* after treatment ($P < 0.025$). *Indicates patients previously treated with tamoxifen.

there may be no statistically significant change in mean DNA polymerase α activity before therapy compared with after, individual patients may show large changes. Clinical response rates to different endocrine therapies such 4-OH A and MPA are typically 30–40% (Harmsen & Porsius, 1988).

A 4-OH A is thought to exert its antiproliferative action by causing a reduction in oestrogen production. Competitive inhibition of aromatase activity in human placental microsomes and tumour regression of hormone dependent carcinogen induced rat tumours following 4-OH A has been demonstrated (Brodie *et al.*, 1983). In postmenopausal women a single injection of 4-OH A (500 mg i.m.) suppressed serum oestradiol to an average of 36% of the base line after 4–7 days (Dowsett *et al.*, 1987).

High dose MPA ($>500\,mg\,day^{-1}$) has been used with some success in the treatment of breast cancer (Izuo *et al.*, 1981) and with other therapies (Ghilchik *et al.*, 1987). *In vitro* MPA has been shown to have an antiproliferative action on breast cancer cell growth and this was accompanied by a reduction in DNA polymerase α activity (Purohit *et al.*, 1990). The growth inhibitory action of MPA is thought to be mediated through the progestogen receptor (Horwitz *et al.*, 1985), but interactions of MPA with other steroid receptors have been reported (Teulings *et al.*, 1980; Poulin *et al.*, 1989).

We found no significant correlation between oestrogen receptor binding and DNA polymerase α activity in untreated malignant tumours. A negative correlation between malignant breast tumour cell proliferation and oestrogen receptor binding has been reported (Meyer *et al.*, 1986) but the correlation was of a low order. We did observe a significantly higher activity of DNA polymerase α activity in oestrogen receptor negative than oestrogen receptor positive malignant breast tumours. A higher rate of breast tumour proliferation, assessed by flow cytometry, has been found in oestrogen receptor negative than oestrogen receptor positive tumours (Kute *et al.*, 1981). No significant relationship between tumour size and DNA polymerase α was found in our study, however, a weak positive correlation between breast tumour size and tumour cell proliferation has been observed (Meyer *et al.*, 1986).

We thank the CRC and AICR for their financial support; we are also indebted to the staff of St Charles Hospital for their assistance.

### References

APOSHIAN, H.V. & KORNBERG, A. (1962). Enzymatic synthesis of deoxyribonucleic acid. *J. Biol. Chem.*, **237**, 519.

BARR, R.D., SARIN, P., SARNA, G. & PERRY, S. (197). The relationship of DNA polymerase to cell cycle stage. *Eur. J. Cancer*, **12**, 705.

BRODIE, A.M.H., GARRETT, W.M., HENDRICKSON, J.R., TSAI-MORRIS, C.H. & WILLIAMS, J.G. (1983). Aromatase inhibitors, their pharmacology and application. *J. Steroid Biochem.*, **19**, 53.

CHIU, R.W. & BARIL, E.F. (1975). Nuclear DNA polymerases and the HeLa cell cycle. *J. Biol. Chem.*, **250**, 7951.

DEPHILIP, R.M., LYNCH, W.E. & LIEBERMAN, I. (1977). Nuclear DNA polymerases of human carcinomas. *Cancer Res.*, **37**, 702.

DOWSETT, M., GOSS, P.E., POWLES, T.J. & 4 others (1987). Use of the aromatase inhibitor 4-hydroxyandrostenedione in postmenopausal breast cancer: optimisation of therapeutic dose and route. *Cancer Res.*, **47**, 1957.

EDWARDS, D.P., MURTHY, S.R. & MCGUIRE, W.L. (1980). Effects of estrogen and antiestrogen on DNA polymerase in human breast cancer. *Cancer Res.*, **40**, 1722.

FOSTER, D.N. & GURNEY, T. (1976). Nuclear location of mammalian DNA polymerase activities. *J. Biol. Chem.*, **251**, 7893.

GHILCHIK, M.W., SHAIKH, N.A., BERANEK, P.A. & REED, M.J. (1987). Cyclical sequential hormochemotherapy in advanced breast cancer. *Br. Med. J.*, **295**, 1172.

HARMSEN, H.J. & PORSIUS, A.J. (1988). Endrocrine therapy of breast cancer. *Eur. J. Cancer Clin. Oncol.*, **24**, 1099.

HARRIS, J. & GORSKI, J. (1978). Estrogen stimulation of DNA dependent DNA polymerase α activity in immature rat uterus. *Mol. Cell Endrocrinol.*, **10**, 293.

HORWITZ, K.B., WEI, L.L., SEDLACEK, S.M. & D'ARVILLE, C.N. (1985). Progestin action and progesterone receptor structure in human breast cancer: a review. *Rec. Prog. Horm. Res.*, **41**, 249.

HUBSCHER, U. (1983). DNA polymerases in prokaryotes and eukaryotes: mode of action and biological implications. *Experientia*, **39**, 1.

IZUO, M., IINO, Y. & ENDO, K. (1981). Oral high-dose medroxyprogesterone acetate (MAP) in treatment of advanced breast cancer. *Breast Cancer Res. Treat.*, **1**, 125.

KUTE, T.E., MUSS, H.B., ANDERSON, D. & 4 others (1981). Relationship of steroid receptor, cell kinetics, and clinical status in patients with breast cancer. *Cancer Res.*, **41**, 3524.

LOWRY, O.H., ROSEBROUGH, N.J., FARR, L.A. & RANDALL, R.J. (1951). Protein measurement with the Folin phenol reagent. *J. Biol. Chem.*, **193**, 265.

LYNCH, W.E., SHORT, J. & LIEBERMAN, I. (1976). The 7.1S nuclear DNA polymerase and DNA replication in intact liver. *Cancer Res.*, **36**, 901.

MEYER, J.S., PREY, M.U., BABCOCK, D.S. & MCDIVITT, R.W. (1986). Breast carcinoma cell kinetics, morphology, stage, and host characteristics. *Lab. Invest.*, **54**, 41.

NICHOLSON, R.I., SYNE, J.S., DANIEL, C.P. & GRIFFITHS, K. (1979). The binding of tamoxifen to oestrogen receptor proteins under equilibrium and non equilibrium conditions. *Eur. J. Cancer*, **15**, 317.

NIEDBALSKI, W., ZWIERZCHOWSKI, L. & WASILEWSKA, L.D. (1986). DNA polymerases of rabbit mammary gland: Partial purification, characterisation and changes in DNA polymerase activities as a function of physiological state. *Int. J. Biochem.*, **18**, 637.

POULIN, R., BAKER, D., POIRIER, D. & LABRIE, F. (1989). Androgen and glucocorticoid receptor-mediated inhibition of cell proliferation by medroxyprogesterone acetate in ZR-75-1 human breast cancer cells. *Breast Cancer Res. Treat.*, **13**, 161.

PUROHIT, A., LAI, L.C. & 5 others (1990). The effect of medroxyprogesterone acetate on aromatase and DNA polymerase activities in breast tumours. *J. Steroid Biochem.*, **34**, 443.

RENNIE, P.S., SYMES, E.K. & MAINWARING, W.I.P. (1975). The androgenic regulation of the activities of enzymes engaged in the synthesis of deoxyribonucleic acid in the ventral prostate gland. *Biochem. J.*, **152**, 1.

SINCOCK, A.M. (1986). Semiautomated measurement of rapidly hydrolysed DNA in the diagnosis of mammary carcinoma. *Cancer*, **57**, 1.

TEULINGS, F.A.G., VAN GILSE, H.A., HENKELMAN, M.S. & 2 others (1980). Estrogen, androgen, glucocorticoid, and progesterone receptors in progestin-induced regression of human breast cancer. *Cancer Res.*, **40**, 2557.